# Effects of endotoxin exposure on childhood asthma risk are modified by a genetic polymorphism in ACAA1

**DOI:** 10.1186/1471-2350-12-158

**Published:** 2011-12-08

**Authors:** Joanne E Sordillo, Sunita Sharma, Audrey Poon, Jessica Lasky-Su, Kathleen Belanger, Donald K Milton, Michael B Bracken, Elizabeth W Triche, Brian P Leaderer, Diane R Gold, Augusto A Litonjua

**Affiliations:** 1 Channing Laboratory, Department of Medicine, Brigham and Women's Hospital, Boston, MA, USA; 2 Division of Pulmonary and Critical Care Medicine, Department of Medicine, Brigham and Women's Hospital, Boston, MA, USA; 3 Harvard Medical School, Boston, MA, USA; 4 Center for Perinatal, Pediatric and Environmental Epidemiology, Department of Epidemiology and Public Health, Yale University School of Medicine, New Haven, CT, USA; 5 Maryland Institute for Applied Environmental Health, School of Public Health, University of Maryland, College Park, MD, USA; 6 Department of Environmental Health, Harvard School of Public Health, Boston, MA, USA; 7 Brown University School of Medicine, Department of Community Health and Epidemiology, Providence, RI, USA

## Abstract

**Background:**

Polymorphisms in the endotoxin-mediated TLR4 pathway genes have been associated with asthma and atopy. We aimed to examine how genetic polymorphisms in innate immunity pathways interact with endotoxin to influence asthma risk in children.

**Methods:**

In a previous analysis of 372 children from the Boston Home Allergens and the Connecticut Childhood Asthma studies, 7 SNPs in 6 genes (CARD15, TGFB1, LY96, ACAA1, DEFB1 and IFNG) involved in innate immune pathways were associated with asthma, and 5 SNPs in 3 genes (CD80, STAT4, IRAK2) were associated with eczema. We tested these SNPs for interaction with early life endotoxin exposure (n = 291), in models for asthma and eczema by age 6.

**Results:**

We found a significant interaction between endotoxin and a SNP (rs156265) in ACAA1 (p = 0.0013 for interaction). Increased endotoxin exposure (by quartile) showed protective effects for asthma in individuals with at least one copy of the minor allele (OR = 0.39 per quartile increase in endotoxin, 95% CI 0.15 to 1.01). Endotoxin exposure did not reduce the risk of asthma in children homozygous for the major allele.

**Conclusion:**

Our findings suggest that protective effects of endotoxin exposure on asthma may vary depending upon the presence or absence of a polymorphism in ACAA1.

## Background

Exposure to endotoxin, a component of the gram negative bacterial cell wall, has been associated with a lower prevalence of asthma symptoms, and a decreased risk of allergic sensitization in childhood [1-3]. Children exposed to the farming environment, where microbial exposures are high, have lower rates of asthma and allergies [4,5]. The ability of endotoxin to modulate innate immune response may explain its protective effects. Endotoxin binds to the TLR4 receptor on antigen presenting cells, initiating, through an MYD88 dependent pathway, the production of IL-12. Secretion of IL-12 promotes the differentiation of naïve T cells into Th1 cells that blunt the Th2 response implicated in asthma and allergic disease [6]. T-regulatory cell function is enhanced in the presence of endotoxin, suggesting an alternative protective mechanism for this gram-negative bacterial component [6,7]. The effects of environmental endotoxin on allergic disease most likely depend upon timing, dose and duration of exposure [8]. Genetics also play an important role in determining an individual's response to endotoxin. In fact, dose-response curves for endotoxin and allergic disease risk have been shown to vary markedly depending upon the presence or absence of polymorphisms in innate immune genes [9].

Thus far, gene by environment interaction studies examining the impact of genetic polymorphisms on the effects of endotoxin exposure have focused mainly on SNPs in individual candidate genes, including CD14, TLR4, and TLR2. For instance, endotoxin's protective effects are modified by the presence of SNPs in CD14, a soluble factor required for endotoxin's binding to TLR4 [10-12]. For individuals with a polymorphism in TLR4 (+4434), high endotoxin exposure confers protective effects against atopy [13]. In controlled exposure studies, missense polymorphisms in TLR4 (Asp299Gly or Thr399Ile) are associated with differences in airway responsiveness to endotoxin [14]. Genetic polymorphisms in TLR2 may decrease the risk of asthma and atopy in children exposed to farming environments, where microbial exposures are high [13].

While the majority of gene by environment interaction analyses have focused on polymorphisms in these single genes of interest, we considered multiple candidate genes in the endotoxin-TLR binding pathway. Polymorphisms associated with asthma and eczema in our cohort have been reported previously [15], and are tested here for interaction with early-life endotoxin exposure.

## Methods

### Study populations

#### Boston Home Allergens and Asthma Study

Subjects were recruited as part of a metropolitan Boston prospective birth cohort study to investigate the relationships between indoor allergen exposure and the development of asthma and other allergic diseases. Details of the study design have been previously described [16,17]. Between September 1994 and June 1996, 505 infants from 499 families were recruited after delivery at the Brigham and Women's Hospital in Boston, Massachusetts. Inclusion criteria for the study were maternal age ≥ 18 years, residence considered safe without intention to move in the next 12 months, maternal ability to speak English or Spanish, residence within Route 128 (within the metropolitan area), and parental report of a doctor's diagnosis of asthma, hay fever, or allergy to inhaled allergens. Children who were born prematurely (< 36 weeks), had a major congenital anomaly, or were hospitalized in the neonatal intensive care unit after delivery were excluded from the study. Approval for this analysis was obtained from the Institutional Review Board (IRB) of the Brigham and Women's Hospital. Informed consent was obtained from the child's primary caretaker at the time of the first home visit. For subjects recruited into the Boston Home Allergens and Asthma Study, blood samples were collected at age 2-3 years, and again at 4-5 years of age. Questionnaires investigating the subject's home environmental characteristics (i.e. home dampness, smoking, and rugs/carpets) and other relevant exposures (i.e. daycare attendance, other siblings in the home, and presence of pets) were obtained at the first home visit at age 2-3 months and then annually thereafter.

#### Connecticut Childhood Asthma Study

Between September 1996 and December 1998, 1002 families were recruited to participate in the Connecticut Childhood Asthma Study. High risk families were recruited after having had a child at one of five Connecticut hospitals or a hospital in south central Massachusetts. To be eligible, a family had to have a newborn infant (index child) with a sibling less than 11 years old with physician diagnosed asthma. Study design and primary outcomes of the study have been previously published [18,19]. Questionnaires on home characteristics and other relevant exposures were administered at first home visit, quarterly for the first three years, and then annually thereafter. Approval was obtained from the IRB of each participating institution and informed consent was obtained from the mother of the study participant prior to entry.

#### Early Life Endotoxin Exposure

Early life exposure to lipopolysaccharide (LPS) from gram negative bacteria was assessed in both cohorts at age 2-3 mths. We collected dust samples from infant's homes by vacuuming both the family room floor and an upholstered chair most often used by the parent while holding the infant. For the Boston Cohort, the biological activity of LPS (endotoxin) was measured in house dust samples using the kinetic Limulus Amoebocyte Lysate (LAL) assay. In the Connecticut Cohort, 3-hydroxy fatty acids (3- OHFAs), biomarkers of LPS that correlate with endotoxin, were measured by GC/MS. Details of the kinetic Limulus Amoebocyte Lysate and the GC/MS method for 3-OHFAs are described elsewhere [20,21]. In the Connecticut Cohort, a small sub-study was conducted to compare endotoxin levels by LAL with levels of 3-OHFAs by GC/MS. Levels of the 3-OHFA C14:0 showed the highest correlation with LAL endotoxin levels (r = 0.9). Exposure to lipopolysaccharide (endotoxin or C14:0 3-OHFA) was ranked by quartile within each cohort. This four-level ranking variable (with 1^st ^quartile as reference) was entered into statistical models as an indicator of endotoxin exposure. There were 291 children with endotoxin exposure assessment, SNP genotyping data, and health outcome data.

#### Measurement of total serum IgE

Serum samples from a subset of children from both cohorts were analyzed for total IgE levels by using an enzyme immunoassay based on the sandwich technique (UNICAP system; Pharmacia Diagnostics, Kalamazoo, Mich). A total of 272 children had available serum IgE values for this analysis. All IgE values were converted to the log-natural scale for analysis.

#### Genotyping and Identification of SNPs associated with Asthma

Candidate genes were selected on the basis of their involvement in the endotoxin and innate immune pathways. SNPs in 44 candidate genes were selected for investigation if they were (1) tagging SNPs with r^2 ^< 0.80 with a minor allele frequency > 5%, covering 10 kb upstream and downstream of the first and last exons of each gene, (2) non-synonymous SNPs resulting in an amino acid change with minor allele frequency> 1%, or (3) variants that had been previously associated with either asthma or asthma-related phenotypes [15]. SNP genotyping was performed using the Illumina BeadStation 500G (San Diego, CA, USA). If a significant interaction was detected for SNPs flanking the exonic regions of a candidate gene, then supplemental genotyping was done to include more SNPs in the flanking region, as well as additional SNPs in the candidate gene.

The Boston Home Allergens and Asthma and the Connecticut Childhood Asthma Study cohorts were similar with respect to their geographic ascertainment area, recruitment scheme, data collection time frames, and data collection methods. In a similar manner to previous work, the two cohorts were combined for this analysis [15,17]. No evidence of population stratification was detected in the combined cohort (p-value = 0.90). (A total of 100 unlinked SNPs were genotyped in all subjects and were analyzed according to the method proposed by Pritchard and Rosenberg [22]). Population stratification SNPs were chosen from the Celera dataset on the TSC website http://snpdata.cshl.edu that were included in the stratification panel, had a minor allele frequency ≥ 0.25 in Caucasians, were greater than 100 kb apart, and did not map to a gene in SNPper http://snpper.chip.org/. SNPs chosen had Illumina scores ≥ 0.65, indicating a high likelihood of successful genotyping.

Linkage disequilibrium patterns were similar for the two cohorts [17]. Due to inadequate sample size of other ethnic groups, only Caucasian subjects were analyzed in this study. Asthma affection status was established using parental report of a doctors' diagnosis of asthma. Children who had physician diagnosed asthma during the first 6 years of life were defined as cases, while the others were classified as non-asthma controls. Furthermore, children who had a report of eczema during the first 6 years of life were defined as cases and those without were defined as non-eczema controls. Association testing was performed using the case control based association testing (C2BAT) methodology. Seven SNPs in 6 genes (ACAA1,TGF-β, DEF-β1, LY96, CARD15, IFN-γ) were associated with asthma (Additional file 1 Table S1), and 5 SNPs in 3 genes (CD80, STAT4, IRAK2) were associated with eczema (Additional file 2 Table S2). We tested the interaction of these SNPs with endotoxin exposure level in multiple regression models, to determine if the effects of exposure were modified by these genetic polymorphisms.

#### Statistical Analyses

To test for interactions between endotoxin exposure and genetic polymorphisms in innate immune genes, we entered the main effect of the SNP and endotoxin exposure, along with a multiplicative interaction term (SNP × endotoxin exposure) into models for the development of the disease outcome (asthma or eczema) by age 6. We used logistic regression for these analyses. For the asthma outcome, seven multiple regression models were constructed (one for each SNP associated with asthma in the previous genetic association analysis), assuming a dominant genetic model. (Although the previous genetic association analysis assumed an additive genetic model, we used a dominant genetic model to increase the power to detect gene by environment interactions). Models were adjusted for potential confounders, including maternal asthma, day care (1-6 mths), low income, breastfeeding, sex and cohort. We used Bonferroni correction (0.05/7 tests) to calculate a p-value threshold for significance adjusted for multiple comparisons (p < 0.007). The same modeling procedure was conducted for the eczema by age 6 outcome, using 5 a priori SNPs (associated with eczema in a previous analysis), endotoxin exposure by quartile, and the multiplicative interaction term (SNP × endotoxin exposure). Potential confounders, including maternal eczema, day care (1-6 mths), low income, breast feeding, sex and cohort were controlled for in the eczema models.

Additionally, we examined total IgE (converted to natural log scale) at age 2 as an outcome variable. Samples assayed for IgE with undetectable levels were assigned a value of 0.175 kU/L (one half the limit of detection for the assay). The main effects of endotoxin exposure and genetic polymorphisms (SNPs previously associated with asthma or eczema) were entered into multiple regression models for total IgE, along with an interaction term (SNP × endotoxin quartile).

## Results

### Population Characteristics

The Boston Home Allergens and Asthma Cohort and Connecticut Childhood Asthma Cohort were comparable in recruitment scheme, geographic ascertainment, and demographic characteristics, as previously described. Therefore, we were able to combine the two cohorts, enhancing our power to detect gene by environment interactions. Of the 291 children with available data on endotoxin exposure, genotyping and health outcomes, 95 were identified as asthma cases, and 196 were controls who did not have asthma. Asthmatic children were more likely to be boys, and were more likely to have a maternal history of asthma or eczema. In all other respects, asthmatic children were similar to controls (Table 1). Approximately half of the asthma cases and controls came from each cohort (48 asthma cases and 100 controls from the Connecticut cohort; 47 asthma cases and 96 controls from the Boston cohort). In the Connecticut cohort, there were 89 eczema cases and 51 controls; in the Boston cohort, 73 cases and 67 controls. The percentage of eczema cases from the Connecticut cohort was higher (55% of total eczema cases) than the percentage of eczema cases (45%) from the Boston cohort (p = 0.053 for Chi Square). Although a subset of Caucasian subjects had genotyping, the characteristics of this subset (sex, maternal/paternal history of asthma and eczema, day care attendance, and pet ownership) were similar to those for the total number of Caucasian subjects enrolled in the Boston (Additional file 3 Table S3) and Connecticut cohorts (Additional file 4 Table S4).

**Table 1:** Baseline Characteristics of Asthma Cases and Controls

Variable	Asthma Cases	Asthma Controls
Subjects (with DNA and endotoxin exposure)	95	196
Gender (male)*	59/95 (0.62)	101/196 (0.51)
Asthmatic mother*	39/95 (0.41)	44/196 (0.22)
Asthmatic father	8/95 (0.08)	15/196 (0.08)
Mother with eczema*^,^	34/95(0.36)	43/196 (0.22)
Father with eczema	13/95 (0.14)	21/196 (0.11)
Attended day care for the first six months of life	30/95 (0.32)	57/196 (0.29)
Income Level:		
< 30,000	6/95 (0.06)	12/196 (0.06)
30,000 to 50000	12/95 (0.13)	25/196 (0.13)
> 50,000	75/95 (0.79)	155/196 (0.79)
Ever Breastfed	75/95 (0.79)	152/196 (0.78)
Eczema diagnosis before age 6*	67/95 (0.71)	95/196 (0.48)

*Covariate varies significantly between asthma cases and asthma controls (p < 0.05)

### Endotoxin Exposure

Exposure to LPS, assessed by either C14:0 3-OHFA (Connecticut Cohort) or by LAL endotoxin (Boston Cohort), was right skewed. In the Boston cohort, the median endotoxin level was 80 and the inter-quartile range was 52 to 126 EU/mg dust. In the Connecticut cohort, the median C14:0 3-OHFA level was 49 and the inter-quartile range was 36 to 81 pmoles/mg dust. In order for the exposure metrics to be comparable, endotoxin/C14:0 3-OHFA levels were ranked according to quartile. Home characteristics associated with increased microbial biomarker levels (pet ownership and dampness) were also similar between the cohorts.

### Gene by Environment Interactions

**Figure 1:**
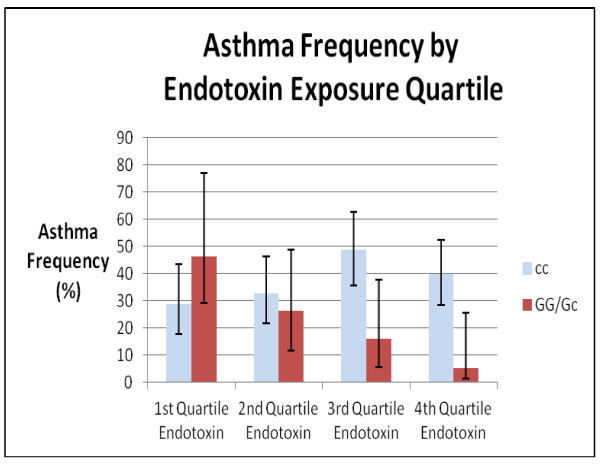
**Interaction between Endotoxin Exposure and SNP (rs156265) in ACAA1 (Asthma frequency by endotoxin quartile and genotype shown with 95% confidence intervals)**.

We tested the 7 SNPs previously identified as predictors of increased asthma risk [15] in models including an interaction term for endotoxin exposure and the SNP of interest. Of the 7 SNPs tested in logistic regression models, rs156265 (ACAA1) interacted with endotoxin exposure to decrease asthma risk (Figure 1). The p value for this interaction term was p = 0.003, which was significant after adjusting for multiple comparisons, and remained so even after adjusting for potential confounders (breastfeeding, daycare attendance, income, maternal asthma, sex and cohort) (p = 0.0013) (Table 2). The effect of cohort (Connecticut/Boston) was not significant in models for asthma, and did not alter the observed interaction between endotoxin exposure and rs156265 SNP.

**Table 2:** Early life endotoxin exposure and asthma by age 6: Effect modification by genetic polymorphisms in the endotoxin/TLR signaling pathway† (P values for interaction term shown)*

Gene	**SNP **†	Genotype	OR (95%CI) for quartile increase in endotoxin	p-value for Interaction (SNP*Endotoxin Quartile)
ACAA1 (near MYD88)	rs156265	CC CG/GG	1.21 (0.92 to 1.57) 0.39 (0.15 to 1.01)	0.0013
LY96	rs16938758	AA AT/TT	1.00 (0.77 to 1.30) 1.16 (0.52 to 2.55)	0.59
DEFB1	rs5743404	TT TC/CC	1.24 (0.83 to 1.85) 0.95 (0.39 to 2.30)	0.28
IFNG	rs2069718	CC CT/TT	1.02 (0.69 to 1.49) 1.02 (0.43 to 2.42)	0.98
CARD15	rs5743291	GG GA/AA	1.09 (0.84 to 1.42) 0.96 (0.41 to 2.25)	0.67
TGFB1	rs12980942	GG GA/AA	1.04 (0.79 to 1.38) 0.96 (0.44 to 2.09)	0.75
TGFB1	rs6957	AA AG/GG	1.04 (0.78 to 1.39) 0.97 (0.45 to 2.09)	0.77

†SNPs tested for interaction were previously associated with asthma in this cohort *Models adjusted for maternal asthma, daycare (1-6 mths), income level, breastfeeding, cohort and sex

**Figure 2:**
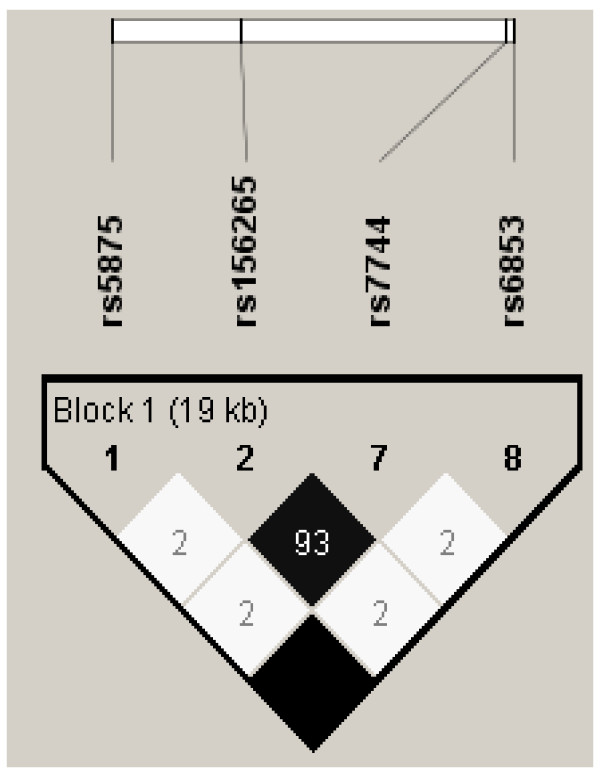
**Linkage Disequilibrium Plot with R^2 ^(HapMap, Caucasians) for SNPs in MYD88 and ACAA1**.

**Figure 3:**
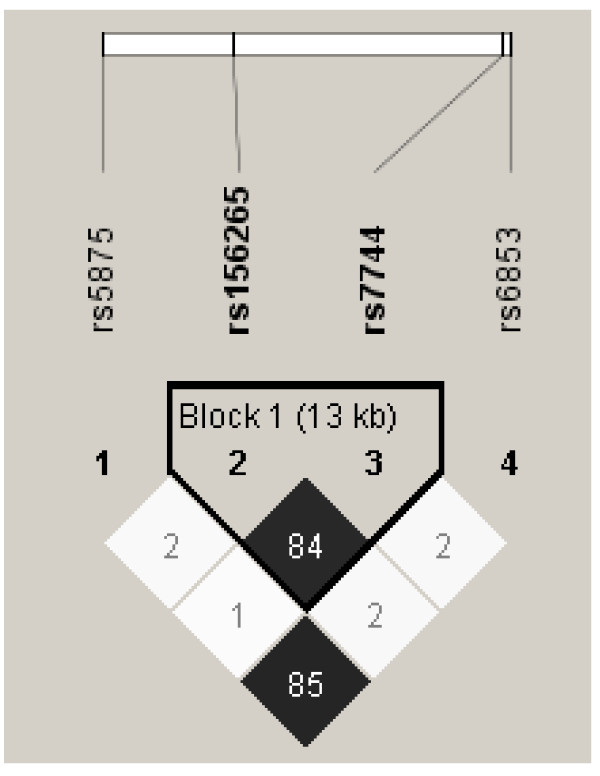
**Linkage Disequilibrium Plot with R^2 ^for SNPs in combined Connecticut Childhood Asthma and Boston Home Allergens Cohorts (Caucasians only)**.

**Figure 4:**
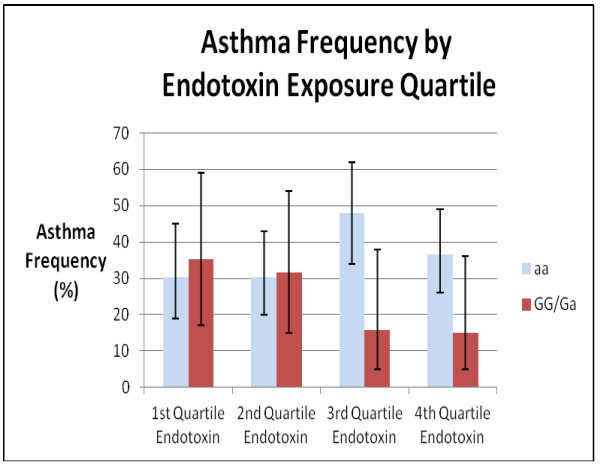
**Interaction between Endotoxin Exposure and SNP (rs7744) in MYD88 (Asthma frequency by endotoxin quartile and genotype shown with 95% confidence intervals)**.

We genotyped this SNP in ACAA1 because it is within 10 kb of MYD88, one of our candidate innate immunity genes. ACAA1 (Acetyl CoA-Acyl Transferase) is an enzyme important for β-oxidation of fatty acids in peroxisomes. To investigate ACAA1 and MYD88 polymorphisms further, we genotyped additional SNPs in both genes, including an MYD88 SNP (rs7744) with the highest R^2 ^value (93%) corresponding to rs156265, as well as two unrelated SNPs in MYD88 (rs6853) and ACAA1 (rs5875). These SNPs were chosen based on the linkage disequilibrium plot for polymorphisms in the two genes generated using the reference HapMap database (Figure 2). Creation of an LD plot using the supplemental SNPs genotyped in the Connecticut/Boston cohorts showed that rs7744 and rs156265 were not as strongly associated (R^2 ^= 84%) as they were in the HapMap reference population (Figure 3). The effects of endotoxin exposure were modified by rs7744 in MYD88 (Figure 4), although the p value for the interaction term (p = 0.03) was not statistically significant after adjustment for multiple comparisons. The other two SNPs in MYD88 and ACAA1 did not modify the effects of endotoxin exposure (Table 3). The effect of endotoxin exposure was only significant in the context of an interaction. Models without the interaction term for rs156265 and endotoxin quartile did not show the protective effect of endotoxin on asthma risk.

**Table 3:** Additional MYD88 and ACAA1 Polymorphisms: Interactions with Endotoxin Exposure and Asthma Risk*

Gene	SNP	Base change	p-value for Interaction (SNP*Endotoxin Quartile)
**ACAA1 (near MYD88)**	rs156265	C > G	0.0013
**MYD88**	rs7744	A > G	0.03
**MYD88**	rs6853	A > G	0.78
**ACAA1**	rs5875	T > A	0.74

*Models adjusted for maternal asthma, daycare (1-6 mths), income level, breastfeeding, cohort and sex

None of the 5 SNPs previously associated with eczema, [15] significantly modified the effect of early life endotoxin exposure on the development of eczema by age 6. Although p values for the interaction terms did not reach statistical significance (p < 0.01) after adjustment for multiple comparisons, two SNPs in CD80 (rs7630595, rs1307124) and one in IRAK2 (rs263408) demonstrated p values of borderline significance (p = 0.02 to 0.03) for the interaction with endotoxin exposure (Table 4). SNPs previously associated with asthma or eczema did not modify the effect of early life endotoxin exposure on total IgE levels (Additional file 5 Table S5).

**Table 4:** Early life endotoxin exposure and Eczema by age 6: Effect modification by genetic polymorphisms in the endotoxin/TLR signaling pathway† (P values for interaction term shown)*

Gene	**SNP**†	Genotype	OR (95%CI) for quartile increase in endotoxin	p-value for Interaction (SNP*Endotoxin Quartile)
CD80	rs7630595	GG GA/AA	1.11 (0.85 to 1.44) 0.57 (0.24 to 1.32)	0.03
IRAK2	rs263408	TT TC/CC	0.87 (0.67 to 1.11) 1.80 (0.74 to 4.37)	0.02
CD80	rs13071247	AA AC/CC	1.14 (0.87 to 1.49) 0.64 (0.29 to 1.40)	0.03
CD80	rs6808536	GG GT/TT	1.08 (0.82 to 1.42) 0.74 (0.34 to 1.61)	0.14
STAT4	rs925847	CC CT/TT	1.07 (0.78 to 1.47) 0.90 (0.42 to 1.94)	0.45

†SNPs tested for interaction were previously associated with eczema in this cohort *Model adjusted for daycare, maternal eczema, breastfeeding, income level, cohort and sex

## Discussion

The results of our study show modification of the association between endotoxin exposure and asthma by a genetic polymorphism in ACAA1, demonstrating that higher home endotoxin levels appear to confer protection against asthma only in those individuals with this SNP (rs156265). This finding is unique, and offers a potential explanation for some of the inconsistent effects of endotoxin exposure observed across multiple epidemiological studies. In other work, investigators have previously shown markedly different dose response curves in response to endotoxin, depending upon the presence or absence of a SNP in CD14 [9]. A cross sectional study of farm children also found that a polymorphism in TLR4 determined whether or not endotoxin was associated with decreased prevalence of atopy in children [13]. Taken together, these studies (and ours) demonstrate the importance of characterizing the underlying genetic susceptibility of individuals before attempting to determine the impact of environmental exposures on allergic disease.

The majority of investigators examining the impact of genetics on responses to environmental microbial exposures in childhood asthma have focused on polymorphisms in CD14, TLR4, and TLR2. Our candidate gene approach allowed us to scan polymorphisms in and around multiple innate immune genes involved in the endotoxin-TLR signaling pathway. By testing a broader range of polymorphisms in biologically relevant genes, we were able to capture an interaction with a polymorphism in ACAA1 that would not have been observed using the single gene approach (TLR2, TLR4, or CD14) commonly employed in gene by environment interaction studies.

While we were able to detect effect modification of endotoxin exposure by this previously unstudied ACAA1 polymorphism (rs156265) we did not observe significant main effects [15] or interactions for the classically studied genes (CD14, TLR4, TLR2) related to endotoxin/TLR signaling. One potential explanation for this is that other polymorphisms, such as those in and around the cell signaling molecule MYD88, may play a more important role in endotoxin mediated responses than CD14, TLR2 or TLR4. (Other studies have not queried this many genes in the pathway, and therefore may have overlooked the importance of these other signaling molecules). It is also possible that effect modification by genetic polymorphisms may vary depending upon the endotoxin levels experienced by the cohort. The majority of gene by environment studies on endotoxin exposure and allergic disease have focused on farm children, who are exposed to higher levels of endotoxin than in suburban cohorts like ours.

Although ACAA1 is not an innate immune gene involved in endotoxin TLR signaling, we genotyped the rs156265 SNP in this region because of its proximity to MYD88. MYD88 is an adaptor molecule involved in endotoxin-mediated TLR signaling in innate immune cells. The MYD88 dependent signaling cascade ultimately results in the production of IL-12, a cytokine responsible for differentiation of Th1 cells that down-regulate the asthma-promoting Th2 response [6].

The ACAA1 SNP that modified the association between endotoxin and asthma risk is found less than 10 kb from MYD88, in a region that may regulate MYD88 transcription. The potential regulatory effect of ACAA1 on MYD88 will require further investigation. In addition to possible regulatory effects, ACAA1 may act as part of a gene cluster. Recently, GWAS analyses have begun to identify gene clusters that may play an important role in allergic disease. For example, genetic polymorphisms within a tumor necrosis factor gene cluster on chromosome 6p (including the genes for TNF-α and lymphotoxin α) have been shown to influence asthma and asthma-related phenotypes [23]. ACAA1 could act as part of a similar gene cluster, responsible for altering innate immune function in response to endotoxin.

The rs156265 SNP was not in linkage disequilibrium with any SNPs in the MYD88 gene, suggesting that this polymorphism is an independent factor in modifying response to endotoxin, and not simply a surrogate for a polymorphism in MYD88. The clear dose response observed for the protective effects of endotoxin exposure in those individuals with the ACAA1 SNP strengthens the evidence that this genetic polymorphism alters response to environmental endotoxin, either through regulation of MYD88 or by an alternative mechanism.

While this study clearly demonstrated a gene by environment interaction for a SNP in ACAA1 and environmental endotoxin exposure, there were some study limitations. In order to increase our power to detect gene by environment interactions, we tested only those genetic polymorphisms significantly associated with asthma or eczema (regardless of environmental endotoxin exposure) in our cohort. This approach allowed us to minimize the problem of multiple comparisons; however, we may have missed interactions between endotoxin exposure and genetic polymorphisms that were not significant as main effects in models for asthma or eczema risk. Even after combining two separate cohorts, the total number of children with complete genotype, endotoxin and health outcome data was relatively small. Although we were able to detect a significant gene by environment interaction, sample size may have reduced our power to detect additional interactions between endotoxin and other genetic polymorphisms. The use of high risk cohorts in this study may limit the generalizability of our results to populations that are not high risk. Lastly, we did not have a replication population for this study, which would have provided additional confirmation of our findings.

## Conclusion

Our findings suggest that protective effects of endotoxin exposure on asthma may vary depending upon the presence or absence of a polymorphism in ACAA1.

## Competing interests

The authors declare that they have no competing interests.

## Authors' contributions

KB, DKM, MBB, EWT, BPL, JES, DRG, and AAL were all involved in the study design for this gene by environment interaction analysis. JES and AAL developed the specific data analysis plan together. KB, MBB, EWT and BPL acquired the data for the Yale Childhood Asthma study. AAL and DRG acquired data for the Boston Home Allergens Cohort. DKM's lab conducted endotoxin analysis by LAL, and 3-OHFA analysis by GC/MS. JES wrote the manuscript, and was the primary data analyst. SS and AP provided supplementary help with the data analysis. JLS served as a biostatistics consultant. All authors reviewed and edited the manuscript for scientific content. All authors have read and approved the final manuscript.

## Pre-publication history

The pre-publication history for this paper can be accessed here:

http://www.biomedcentral.com/1471-2350/12/158/prepub

## Supplementary Material

Additional File 1 **Table S1: (Sharma et al, in press) Associations between genetic polymorphisms and asthma by age 6**. Table from Sharma et al manuscript, accepted for publication by Pediatric Allergy and Immunology. Click here for file

Additional File 2 **Table S2: (Sharma et al, in press) Association between genetic polymorphisms and eczema by age 6**. Table from Sharma et al manuscript, accepted for publication by Pediatric Allergy and Immunology. Click here for file

Additional File 3 **Table S3: Comparison of Boston Home Allergens study subjects with DNA vs. those without DNA**. Click here for file

Additional File 4 **Table S4: Comparison of Connecticut Childhood Asthma Study subjects with DNA vs. those without DNA**. Click here for file

Additional File 5 **Table S5: Early Life Endotoxin exposure and total IgE in early childhood: Effect of modification by genetic polymorphisms in the endotoxin TLR signaling pathway**. Click here for file
