# Supplementary material for: Effects of endotoxin exposure on childhood asthma risk are modified by a genetic polymorphism in ACAA1
Source: BMC Med Genet. 2011 Dec 8;12:158. doi: 10.1186/1471-2350-12-158 (PMC3252252; doi:10.1186/1471-2350-12-158)
Supplement: Additional File 1 — Table S1: (Sharma et al, in press) Associations between genetic polymorphisms and asthma by age 6. Table from Sharma et al manuscript, accepted for publication by Pediatric Allergy and Immunology. [file 1471-2350-12-158-S1.DOC]

| **Gene** | **SNP** | **Base change** | **Minor allele frequency** | **Case genotype frequencies (AA, Aa, aa)** | **Control genotype frequencies (AA, Aa, aa)** | **OR** | **95% CI** | **p-value** |
| --- | --- | --- | --- | --- | --- | --- | --- | --- |
| TGFB1 | rs6957 | A>G | 0.18 | 69 (0.61), 37 (0.32), 8 (0.07) | 175 (0.73), 55 (0.23), 9 (0.04) | 1.8 | 1.21-2.67 | 0.0037 |
| DEFB1 | rs5743404 | T>C | 0.38 | 35 (0.30), 57 (0.50), 23 (0.20) | 104 (0.42), 115 (0.47), 28 (0.11) | 1.57 | 1.12-2.19 | 0.0083 |
| TGFB1 | rs12980942 | G>A | 0.15 | 74 (0.66), 32 (0.29), 6 (0.05) | 184 (0.76), 54 (0.22), 5 (0.02) | 1.78 | 1.16-2.74 | 0.0086 |
| ACAA1 | rs156265 | C>G | 0.15 | 91 (0.82), 19 (0.17), 1 (0.01) | 164 (0.70), 64 (0.27), 2 (0.03) | 0.52 | 0.31-0.87 | 0.0123 |
| LY96 | rs16938758 | A>T | 0.17 | 88 (0.77), 27 (0.23), 0 (0.00) | 159 (0.65), 79 (0.32), 8 (0.03) | 0.55 | 0.34-0.89 | 0.0141 |
| CARD15 | rs5743291 | G>A | 0.10 | 86 (0.76), 23 (0.20), 4 (0.04) | 205 (0.84), 37 (0.15), 2 (0.01) | 1.85 | 1.12-3.06 | 0.0158 |
| IFNG | rs2069718 | C>T | 0.42 | 51 (0.45), 40 (0.36), 21 (0.19) | 75 (0.31), 120 (0.49), 49 (0.20) | 0.69 | 0.50-0.95 | 0.0216 |

**Supplemental Table 1. Associations between genetic polymorphisms and asthma by age 6***

*Adjusted for paternal asthma, maternal asthma, and daycare attendance during the first six months of life
