# Supplementary material for: Effects of endotoxin exposure on childhood asthma risk are modified by a genetic polymorphism in ACAA1
Source: BMC Med Genet. 2011 Dec 8;12:158. doi: 10.1186/1471-2350-12-158 (PMC3252252; doi:10.1186/1471-2350-12-158)
Supplement: Additional File 2 — Table S2: (Sharma et al, in press) Association between genetic polymorphisms and eczema by age 6. Table from Sharma et al manuscript, accepted for publication by Pediatric Allergy and Immunology. [file 1471-2350-12-158-S2.DOC]

**Supplemental Table 2. Associations between genetic polymorphisms and eczema by age 6***

| **Gene** | **SNP** | **Base change** | **Minor allele frequency** | **OR** | **95% CI** | **p-value** |
| --- | --- | --- | --- | --- | --- | --- |
| CD80 | rs7630595 | G>A | 0.13 | 2.436 | 1.46-4.07 | 0.0007 |
| CD80 | rs6808536 | G>T | 0.17 | 1.749 | 1.13-2.70 | 0.0118 |
| CD80 | rs13071247 | A>C | 0.16 | 1.753 | 1.13-2.72 | 0.0129 |
| STAT4 | rs925847 | C>T | 0.28 | 0.633 | 0.44-0.91 | 0.0137 |
| IRAK2 | rs263408 | T>C | 0.1 | 1.966 | 1.13-3.41 | 0.0166 |
| *Adjusted for paternal eczema, maternal eczema, and daycare attendance during the first six months of life | | | | | | |
