# Supplementary material for: Effects of endotoxin exposure on childhood asthma risk are modified by a genetic polymorphism in ACAA1
Source: BMC Med Genet. 2011 Dec 8;12:158. doi: 10.1186/1471-2350-12-158 (PMC3252252; doi:10.1186/1471-2350-12-158)
Supplement: Additional File 3 — Table S3: Comparison of Boston Home Allergens study subjects with DNA vs. those without DNA. [file 1471-2350-12-158-S3.DOC]

**Supplemental Table 3. Comparison of Boston** Home Allergens Subjects without DNA vs. those with DNA

|  | Caucasian Home Allergens Subjects without DNA (n=192) | Caucasian Home Allergens Subjects with DNA (n=183) | P value* |
| --- | --- | --- | --- |
| Gender (male) | 105 (55%) | 101 (55%) | 0.9 |
| Asthmatic mother | 47 (24%) | 49 (27%) | 0.6 |
| Asthmatic father *(6 missing)* | 47 (24%) | 37 (20%) | 0.3 |
| Mother with eczema *(5 missing)* | 46 (24%) | 38 (21%) | 0.4 |
| Father with eczema *(11 missing)* | 31 (17%) | 21 (12%) | 0.2 |
| Attended day care for the first six months of life | 68 (35%) | 70 (38%) | 0.6 |
| Attended day care between months 7 to 12 after birth | 15 (8%) | 22 (12%) | 0.2 |
| Dog in home - year 1 | 32 (17%) | 38 (21%) | 0.3 |
|  |  |  |  |
| *Comparison for those with DNA vs. those without DNA |  |  |  |
