# Supplementary material for: Effects of endotoxin exposure on childhood asthma risk are modified by a genetic polymorphism in ACAA1
Source: BMC Med Genet. 2011 Dec 8;12:158. doi: 10.1186/1471-2350-12-158 (PMC3252252; doi:10.1186/1471-2350-12-158)
Supplement: Additional File 4 — Table S4: Comparison of Connecticut Childhood Asthma Study subjects with DNA vs. those without DNA. [file 1471-2350-12-158-S4.DOC]

**Supplemental Table 4. Comparison of Connecticut Childhood Asthma Study (CHAS) Subjects without DNA vs. those with DNA**

|  |  |  |  |
| --- | --- | --- | --- |
|  | Caucasian CHAS Subjects without DNA (n=279) | Caucasian CHAS Subjects with DNA (n=270) | P value* |
| Gender (male) | 140 (52%) | 130 (48%) | 0.52 |
| Asthmatic mother | 183(67%) | 87(32%) | 0.1 |
| Asthmatic father *(4 missing)* | 225(84%) | 42(15%) | 0.3 |
| Mother with eczema *(81 missing)* | 185 (71%) | 77(29%) | 0.95 |
| Father with eczema *(95 missing)* | 215(84%) | 40 (16%) | 0.9 |
| Attended day care for the first six months of life (*23 missing*) | 213 (80%) | 54 (20%) | 0.6 |
| Attended day care between months 7 to 12 after birth | 192 (72%) | 75(28%) | 0.4 |
| Dog in home - year 1 | 139(60%) | 140(58%) | 0.8 |
|  |  |  |  |
| *Comparison for those with DNA vs. those without DNA |  |  |  |
