# Supplementary material for: Effects of endotoxin exposure on childhood asthma risk are modified by a genetic polymorphism in ACAA1
Source: BMC Med Genet. 2011 Dec 8;12:158. doi: 10.1186/1471-2350-12-158 (PMC3252252; doi:10.1186/1471-2350-12-158)
Supplement: Additional File 5 — Table S5: Early Life Endotoxin exposure and total IgE in early childhood: Effect of modification by genetic polymorphisms in the endotoxin TLR signaling pathway. [file 1471-2350-12-158-S5.DOCX]

**Supplemental Table 5. Early life endotoxin exposure and total IgE in early childhood: Effect modification by genetic polymorphisms in the endotoxin/TLR signaling pathway†**

| **Gene** | **SNP**† | **Base change** | **p-value for Interaction (SNP*Endotoxin Quartile)** |
| --- | --- | --- | --- |
| TGFB1 | rs6957 | A>G | 0.03 |
| DEFB1 | rs5743404 | T>C | 0.18 |
| TGFB1 | rs12980942 | G>A | 0.07 |
| ACAA1 | rs156265 | C>G | 0.53 |
| LY96 | rs16938758 | A>T | 0.26 |
| CARD15 | rs5743291 | G>A | 0.31 |
| IFNG | rs2069718 | C>T | 0.38 |
| CD80 | rs7630595 | G>A | 0.53 |
| IRAK2 | rs263408 | T>C | 0.80 |
| CD80 | rs13071247 | A>C | 0.80 |
| CD80 | rs6808536 | G>T | 0.32 |
| STAT4 | rs925847 | C>T | 0.32 |
